# Tailoring the Magnetic and Structural Properties of Manganese/Zinc Doped Iron Oxide Nanoparticles through Microwaves-Assisted Polyol Synthesis

**DOI:** 10.3390/nano12193304

**Published:** 2022-09-22

**Authors:** Margherita Porru, María del Puerto Morales, Alvaro Gallo-Cordova, Ana Espinosa, María Moros, Francesca Brero, Manuel Mariani, Alessandro Lascialfari, Jesús G. Ovejero

**Affiliations:** 1 Dipartimento di Fisica, Università degli Studi di Pavia, Via A. Bassi 6, 27100 Pavia, Italy; 2 Istituto Nazionale di Fisica Nucleare, Sezione di Pavia, Via A. Bassi 6, 27100 Pavia, Italy; 3 Instituto de Ciencia de Materiales de Madrid, ICMM/CSIC, C. Sor Juana Inés de la Cruz 3, 28049 Madrid, Spain; 4 IMDEA Nanociencia, c/ Faraday, 9, 28049 Madrid, Spain; 5 Nanobiotecnología (IMDEA-Nanociencia) Unidad Asociada al Centro Nacional de Biotecnología (CSIC), 28049 Madrid, Spain; 6 Instituto de Nanociencia y Materiales de Aragón (INMA), CSIC-Universidad de Zaragoza, 50018 Zaragoza, Spain; 7 Centro de Investigación Biomédica en Red de Bioingeniería, Biomateriales y Nanomedicina (CIBER-BBN), 50018 Zaragoza, Spain; 8 Hospital General Universitario Gregorio Marañón, C. Dr. Esquerdo, 46, 28007 Madrid, Spain

**Keywords:** magnetic nanoparticles, Mn and Zn doping, iron oxides, microwave synthesis, polyols technique, NMR, MRI contrast agents

## Abstract

Tuning the fundamental properties of iron oxide magnetic nanoparticles (MNPs) according to the required biomedical application is an unsolved challenge, as the MNPs’ properties are affected by their composition, their size, the synthesis process, and so on. In this work, we studied the effect of zinc and manganese doping on the magnetic and structural properties of MNPs synthesized by the microwave-assisted polyol process, using diethylene glycol (DEG) and tetraethylene glycol (TEG) as polyols. The detailed morpho-structural and magnetic characterization showed a correspondence between the higher amounts of Mn and smaller crystal sizes of the MNPs. Such size reduction was compensated by an increase in the global magnetic moment so that it resulted in an increase of the saturation magnetization. Saturation magnetization M${}_{S}$ values up to 91.5 emu/g and NMR transverse relaxivities r${}_{2}$ of 294 s${}^{-1}$mM${}^{-1}$ were obtained for Zn and Mn- doped ferrites having diameters around 10 nm, whereas Zn ferrites with diameters around 15 nm reached values of M${}_{S}∼$ 97.2 emu/g and of r${}_{2}$∼ 467 s${}^{-1}$mM${}^{-1}$, respectively. Both kinds of nanoparticles were synthesized by a simple, reproducible, and more sustainable method that makes them very interesting for diagnostic applications as MRI contrast agents.

## 1. Introduction

Nanomaterials have been deeply investigated in recent decades because of their versatility in a broad range of biomedical applications, ranging from diagnostic to therapeutic fields. In particular, iron-oxide based magnetic nanoparticles (MNP) are widely investigated as contrast agents for magnetic resonance imaging (MRI) applications [1,2,3] or hyperthermia treatments [4,5,6,7] due to their magnetic and structural characteristics [8,9,10,11]. Ferrites with the general composition MFe${}_{2}$O${}_{4}$, where M is a divalent cation, exhibit a cubic spinel structure in which metallic ions may occupy two interstitial positions, the tetrahedral (T${}_{h}$) and the octahedral (O${}_{h}$) one. The tuning of the size, the chemical composition, and the interstitial occupation in the ferrites offer a wide set of parameters to adjust the magnetic properties of the MNPs to the specific requirements for their biomedical application [12,13,14,15].

The global magnetic moment rises from the balance between the distribution of the ions in both T${}_{h}$ and O${}_{h}$ sublattices: the metallic atoms occupy these interstitial positions, establishing two uncompensated antiparallel magnetic sublattices (ferrimagnetic structure). Common magnetite Fe${}_{3}$O${}_{4}$ has an inverse cubic spinel structure: Fe${}^{3+}$ lies in the T${}_{h}$ site, whereas Fe${}^{2+}$ and Fe${}^{3+}$ are present in the O${}_{h}$ site. The introduction of doping elements, such as zinc and manganese, changes the global magnetic moment of the nanostructures: the non-magnetic ions of zinc tend to replace iron ions in the T${}_{h}$ sites, thus increasing the overall magnetic moment [12,16,17]. The further introduction of manganese ions, with higher atomic moment (MnFe${}_{2}$O${}_{4}$ presents a theoretical magnetic moment of 5μ${}_{B}$), may further enhance saturation magnetization values if they are located in T${}_{h}$ positions [18,19,20]. At the same time, variations in the crystal size of the MNPs are expected, because bigger or smaller ions are introduced into the crystal structure, modifying its lattice parameter but also its surface energy. At the surface level, spins can interact differently than the bulk structure, thus they can present small angle deviations (spin canting) from the co-linear orientation: this non-uniform configuration reduces the MNPs’ magnetization. The distribution of the doping elements in the crystal lattice is strictly linked to their impact on the magnetic and structural properties [17]. The synthesis process of the nanomaterials, the reaction temperature, and the reagents mixture influence the allocation of the ions and the dimensions of the MNPs [21,22].

The microwave-assisted polyol technique employs microwaves (MW) as a heating source and polyols as solvent, reducer, and surfactant concurrently. This method provides a simple and reliable procedure to control the reaction temperature and reduce the reaction times. The microwaves homogeneously heat the reagent mixture, leading to control over the size distribution of the MNPs. In addition, the polyol solvents make it possible to reach high boiling temperatures, similar to organic solvents, but producing hydrophilic nanoparticles that can be easily dispersed in water [23,24,25,26].

This method is cleaner, simpler, and more eco-friendly than other conventional methods, such as thermal decomposition, co-precipitation, or hydrothermal synthesis [27,28]. In addition, it offers a gain in terms of time and energy expenditure, possibly involving non-toxic precursors. In 2016, Hachani et al. [29] studied the polyol, the precursor amount, and the reaction time effects in the polyol synthesis at high pressure and high temperature, parameters that give high control on size and monodispersity of the produced MNPs. The introduction of the MW as a heating source saves time and energy and produces a batch of MNPs with high crystallinity and a narrow size distribution [28]. The rapid heating mechanism, simplicity, and reproducibility of MW-assisted synthesis make this method ideal for the in situ clinical production of advanced MRI contrast agents. However, the effect of polyol nature and reaction temperatures on the occupations and utterly on the magnetic properties of ultrasmall Zn and Mn ferrite nanoparticles for the MW polyol synthesis still remain unexplored.

In this study, two groups of iron oxide based magnetic nanoparticles were produced using diethylene glycol (DEG) and tetraethylene glycol (TEG). For each polyol, a set of zinc-ferrite MNPs were prepared with Zn = 0, 0.2, 0.4, 0.5, 0.8, 1 (DEG), and Zn = 0, 0.3, 0.5, 0.6 (TEG). After selecting the zinc amount that maximizes the magnetic moment of the samples, the composition was further modified by substituting Fe atoms with different amounts of Mn. The advanced occupational and relaxometric studies were focused on the ferrite MNPs of 10 and 15 nm that maximize the saturation magnetization values (MW10ZM and MW15Z) and on two reference samples of magnetite (MW10 and MW15) to compare their efficiency as MRI T${}_{2}$ contrast agents.

## 2. Materials and Methods

### 2.1. Materials

The iron(II) acetate, zinc(II) acetate, and manganese(II) acetate were purchased from Sigma-Aldrich (St. Louis, MO, USA). The diethylene glycol (DEG) and tetraethylene glycol (TEG) solvents were purchased from Sigma-Aldrich as well as the citric acid employed for the coating process. All the reactants were used as received without further purification.

### 2.2. Synthesis of Magnetic Nanoparticles

For the synthesis of iron oxide nanoparticles, 255.7 mg (1.47 mmol) of Fe acetate was mixed with 27 mg (0.15 mmol) of Zn acetate and 19.03 mg (0.11 mmol) of Mn acetate, then blended with 17.3 mL of TEG and 0.7 mL of milliQ water into a 30 mL MW vial and magnetically stirred for 20 min. For the synthesis of Zn and Mn ferrites, the Fe precursor was partially substituted by the metallic precursors in different molar amounts according to the required composition: Mn${}_{x}$Zn${}_{y}$Fe${}_{3-x-y}$O${}_{4}$. The mass of precursors employed for the synthesis of different samples is detailed in Table S1. The heating ramps were carried out in a microwave synthesis reactor Monowave300 (Anton Paar GmbH). The samples were heated up to 230 °C in 1 h (3.83 °C/min), kept at this temperature for 30 min, and then quickly cooled down to 55 °C. The final product was poured into a 50 mL Falcon vial using ethanol to rinse and dilute the solvent to a final volume of 40 mL. The MNPs were washed by centrifugation at 10,000 rpm for 30 min, removing the supernatant and dispersing the residue in ethanol; this process was repeated three times. Finally, the MNPs were dried with N${}_{2}$ to remove any ethanol contamination. The powders were used for magnetic and structural characterization, and part of the sample was dispersed again in water for colloidal characterization, transmission electron microscopy (TEM), and MRI response measurements.

The four selected samples for the MRI response study were colloidally stabilized with a citric acid (CA) coating. For this purpose, 2 mL of a CA solution 0.05 M at pH 4 (regulated with KOH 1 M) was mixed with 1 mL of the uncoated MNPs water dispersion (5 mg/mL). The mixture was then ultrasound stirred for 30 min at 80 ºC. As the obtained product was ultra-stable, samples were purified by dialysis for 2 days to remove unreacted CA using a cellulose membrane tube MWCO 10,000 Da.

### 2.3. Characterization Techniques

*Inductively couple plasma-optical emission spectrometry (ICP-OES)*. The ICP-OES analysis of each sample was performed by Optima 2100 DV PerkinElmer equipment to determine the elemental composition. For this analysis, a small volume of the sample was digested with aqua regia for 24 h at 90 ºC and diluted to a controlled volume.

*X-ray diffraction (XRD)*. The XRD patterns of the samples were acquired with a powder diffractometer Bruker D8 Advance with Cu Kα radiation with an energy-discriminator. The acquisitions were performed on fine powders pressed on a silicon crystal sample holder into the 2θ range [10°; 70°] with an angular step of 0.04°. The pattern was analyzed to accurately evaluate the position and width of the peaks, and, by the Scherrer equation, the crystal size of the MNPs considering the instrumental broadening. The lattice parameters of each sample were extrapolated from the XRD paths by Bragg’s law.

*Transmission electron microscopy (TEM)*. TEM samples were prepared by air-drying a few drops of the colloidal dispersion of MNPs on a carbon-coated copper grid. The TEM pictures were acquired with a 100 keV JEOL-JEM 1010 microscope with a digital camera Gatan model Orius 200 SC (see Figure S1). The images were analyzed with the program ImageJ [30], measuring at least 150 MNPs. The diameters were fitted to a log-normal distribution, and the most probable diameter (see Table S2), with its error, was extrapolated from this distribution.

*Thermogravimetric analysis (TGA)*. The TGA was carried out with approximately 10 mg of powder in a thermal gravimetric analyzer SDT Q600 V20.9 Build 20 under an N${}_{2}$ atmosphere. The percentage of mass loss was used to estimate the amount of inorganic material present in the samples.

*X-ray absorption spectroscopy (XAS)*. X-ray absorption spectra were recorded in transmission mode at CLAESS beamline at ALBA synchrotron (Spain). Spectra at the Fe K-edge (7112 eV) were acquired at room temperature using a Si (311) monochromator at both energy regions (X-ray absorption near edge structure (XANES) and extended X-ray absorption fine structure (EXAFS)). The monochromator energy was calibrated with the K-edge position of an Fe foil. Powdered samples were compressed with cellulose into pellets and each sample was packed between two Kapton films; γ-Fe${}_{2}$O${}_{3}$ and Fe${}_{3}$O${}_{4}$ were chosen as references. Spectra were normalized to the unit edge jump using a linear pre-edge fit and a polynomial post-edge fit for background subtraction. Data were analyzed using the XAS data-analysis program Athena (Ravel and Newville, 2005) [31]. The k${}^{2}$-weighted EXAFS function and Fourier transform (FT) were acquired to obtain the radial distribution around the Fe atoms in the range of 2.5 to 10 Å${}^{-1}$ (not corrected for phase shift).

### 2.4. Magnetic Characterization

*Vibrating sample magnetometer (VSM)*. A weighted mass of powder sample was compacted at the bottom of a capsule with cotton. The weight was used to normalize the magnetic moment measurements into magnetization values after organic percentage correction. The hysteresis loops at 290 K were acquired in a vibrating sample magnetometer MLVSM9 MagLab 9T, Oxford Instrument (see Figure S2), from −5 T to 5 T.

*Nuclear Magnetic Resonance (NMR)*. The relaxometric properties of four selected samples were investigated as a function of the MNPs concentration: the longitudinal and transverse relaxation times (T${}_{1}$ and T${}_{2}$) of the samples were measured on five different iron concentrations in water solutions: 0.10, 0.15, 0.20, 0.25, and 0.30 mM. The NMR acquisitions were done with the Apollo Tecmag (Houston, TX, USA) NMR spectrometer using a static magnetic field of 1.5 T generated by the MAGNET B-E 25, Bruker (Billerica, MA, USA) electromagnet, through the Saturation Recovery (SR) sequence for the T${}_{1}$ acquisitions and the Carr–Purcell–Meiboom–Gill (CPMG) sequence for the T${}_{2}$ ones. The relaxation rates were linearly fitted considering the uncertainties on both relaxation rates and concentrations. This protocol was used to evaluate the relaxivity r${}_{1,2}$ values of the chosen samples, which represent their efficiency as MRI contrast agents. The colloidal stability of the MNPs coated with citric acid was evaluated by measuring at 1.5 T the relaxation rates of 0.15 mM dispersion of each sample in time. Subsequently, the colloidal stability of the former samples was evaluated by measuring once a month for three months each dispersion.

## 3. Results

**Figure 1:**
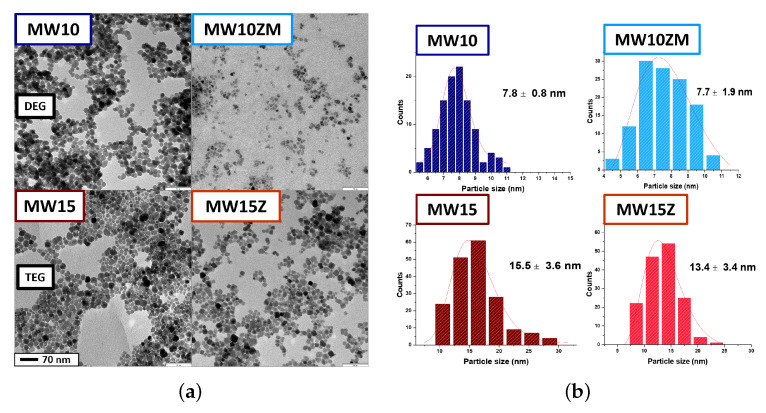
TEM images (**a**) and analysis (**b**) of samples MW10, MW10ZM, MW15, and MW15Z. Samples MW10 and MW10ZM have smaller dimensions than MW15 and MW15Z.

Figure 1 shows the morphological comparison between the MNPs synthesized in diethylene glycol and (DEG) and tetraethylene glycol (TEG) (see Table 1 and Table S2 for the details of the two sets of MNPs). In both cases, the MNPs present a quasispherical shape for iron oxide (MW10 and MW15) and Zn-Mn ferrite (MW10ZM and MW15Z) MNPs. However, the DEG produced smaller nanostructures than those produced using TEG as a result of their different chain lengths. Polyols with longer chains facilitate the growth process of the structure and lead to the production of bigger nanoobjects [29].

**Figure 2:**
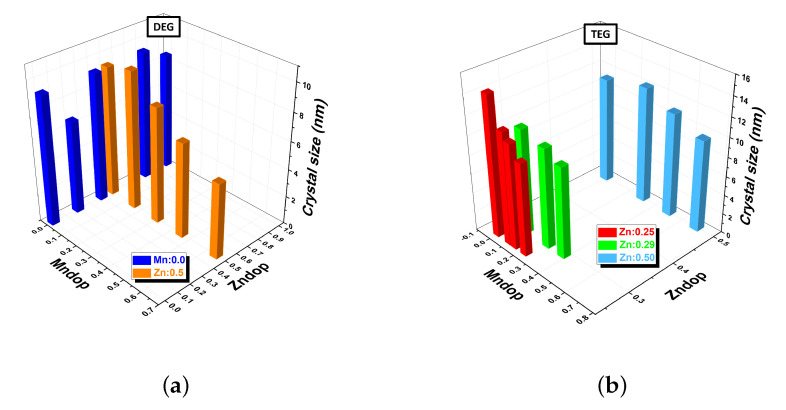
Variation of crystal size as a function of Zn and Mn doping for nanoparticles synthesized with: (**a**) diethylene glycol (DEG) and (**b**) tetraethylene glycol (TEG).

**Figure 3:**
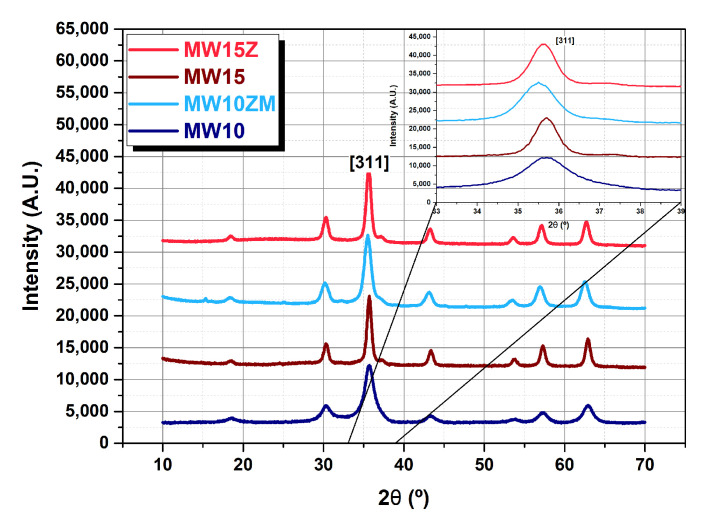
XRD patterns of the samples MW10, MW10ZM, MW15, and MW15Z. Samples MW10 and MW10ZM have smaller dimensions than MW15 and MW15Z.

The effect of Zn and Mn doping on the crystal sizes (diameter) of the MNPs synthesized using DEG and TEG is presented in Figure 2. These crystal sizes (Table S2) were determined from the width of the most intense XRD peak corresponding to the (3 1 1) reflection using the Scherrer equation (Figure 3). Looking at the trends presented in Figure 2, it is clear that doping with manganese reduces the crystal size of the MNPs, regardless of the polyol employed during the synthesis process.

**Figure 4:**
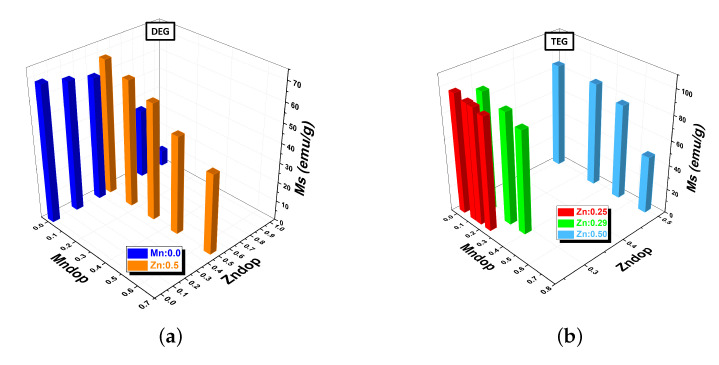
Variation of the saturation magnetization as function of zinc and manganese doping for the samples synthesized with (**a**) DEG and (**b**) TEG.

**Figure 5:**
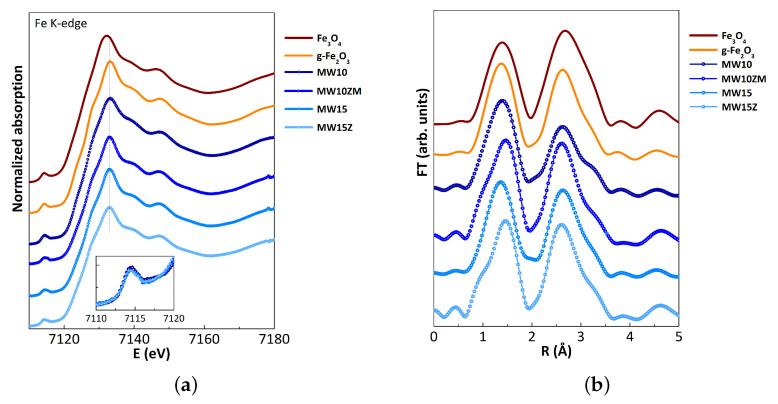
(**a**) Fe K-edge XANES spectra of synthesized samples and iron oxide references from bottom to top. Inset: pre-peak of synthesized samples. (**b**) Fourier transform of k${}^{2}$-weighted EXAFS signal of synthesized samples and iron oxide references (Fe${}_{3}$O${}_{4}$ (magnetite) and γ-Fe${}_{2}$O${}_{3}$ (maghemite)).

The effect of Zn and Mn doping on the saturation magnetization of the MNPs synthesized with both different polyols is presented in Figure 4. The data show that, although the introduction of Mn decreases the crystal size of the synthesized MNPs, it keeps surprisingly high values of saturation magnetization. The surface-to-volume ratio is higher for smaller MNPs, and the spin canting should induce a significant reduction of the saturation magnetization, as observed in the case of iron oxide MNPs (Figure 5). However, it was observed that with the proper conditions of Zn and Mn doping it is possible to compensate for the magnetization reduction produced by spin canting, reaching saturation magnetization values up to 97.2 emu/g.

The four samples presented in Figure 1 were selected among all the compositions explored for a deeper analysis of magnetic properties and MRI contrast efficiency: the MW15Z (γ-Zn${}_{0.24}$Fe${}_{1.76}$O${}_{3}$) was the Zn-doped sample of approximately 15 nm with maximum saturation magnetization; the MW10ZM (γ-Zn${}_{0.26}$Mn${}_{0.23}$Fe${}_{1.56}$O${}_{3}$) was the 10 nm sample doped with Zn and Mn that presents the maximum saturation magnetization value. The two related reference samples of iron oxide (MW10 and MW15) were prepared with similar dimensions but no doping. In this way, it was possible to compare the effect of the doping elements as well as of the MNP size. From the TEM analysis, the resulting diameters of the MNPs are 7.8 ± 0.8 nm for MW10, 7.7 ± 1.9 nm for MW10ZM, 15.5 ± 3.6 nm for MW15, and 13.4 ± 3.4 nm for MW15Z. In Figure 3, the XRD patterns of these selected samples are presented. A progressive shift of the peaks due to the introduction of the doping elements into the spinel structure can be observed. The substitution of iron ions with manganese or zinc ions could dilate or contract the unit cell of the structure. At the same time, when analyzing the peaks spreading, we evaluated the variation of the crystal size (Table 1). In particular, the reference samples MW10 and MW15 have a comparable peak position, whereas the doped ones are shifted to the left, meaning a contraction of the lattice due to interstitial substitution.

For a further inspection of the ferrite doping, the X-ray absorption spectroscopy (XAS) technique was applied to study doping occupancy. This technique provides information about the geometry and charge of the iron ions of ferrite samples and their local structural environment regarding surrounding shells by analyzing the XANES and EXAFS regions of the XAS spectra, respectively. Figure 5a shows the Fe K-edge XANES spectra of the four selected samples together with iron oxide references (Fe${}_{3}$O${}_{4}$ (magnetite) and γ-Fe${}_{2}$O${}_{3}$ (maghemite)). In this region, the edge position allows for assigning the oxidation state of the absorber, Fe in this case. All samples present the same edge position, coincident with that of the maghemite, whose Fe ions are in an average state of Fe${}^{3+}$, in agreement with the spinel structure type in ferrites, where Fe is in 3+ and occupies tetrahedral and octahedral sites. The position of the pre-edge peak is also consistent with the presence of Fe${}^{3+}$ [32]. This pre-edge peak is related to the 1s to 3d transition, forbidden by the dipole selection rules, and its intensity is sensitive to the local coordination geometry of the metal atom [32]. In general, the intensity of the pre-edge peak is lower for Fe${}^{3+}$ octahedral compounds with centrosymmetric sites than that for Fe${}^{3+}$ tetrahedral compounds (with non-centrosymmetric sites) [33]. For the doped ferrites with Zn or Zn and Mn, this pre-edge peak is similar to that of a non-doped compound.

Fourier-filtered k${}^{2}$-weighted EXAFS functions of the synthesized samples and those of iron oxide references are presented in Figure 5b. The selected k-range for the FT spectra was 2.5–10 Å${}^{-1}$. The FT spectra peaks correspond to local atom correlations, and their magnitude is proportional to the number of neighboring atoms around Fe, i.e., Fe-O and Fe-M (M = Fe, Zn, or Mn) bonds. The first coordination peak is similar for all samples, which corresponds to distances of iron in tetrahedral and octahedral sites (Fe-O) around 2.0 Å (not corrected for phase shift). These distances are typical of Fe-based spinel structures such as magnetite, maghemite, or iron-based ferrites, which range from 1.96 to 2.05 Å. Previous structures also present second and third coordination peaks around 3–3.5 Å due to metal–metal distances (Fe–Fe and Fe-M bonds) [23,34]. The reduction of coordination for the Fe–Fe shell is observed for the MW10 sample, which can be attributed to a nanostructure size reduction.

**Figure 6:**
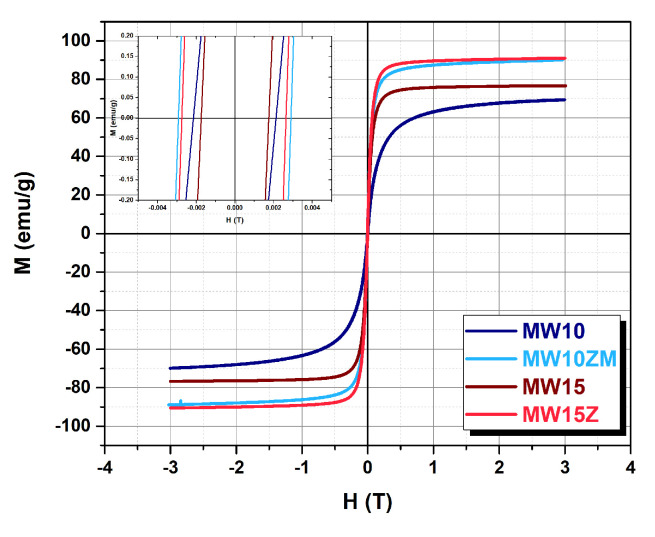
Hysteresis loops at 290 K for samples MW10, MW10ZM, MW15, and MW15Z.

The hysteresis loops of the selected samples, acquired at 290 K, are reported in Figure 6. Their coercive field is below 25 Oe, confirming the superparamagnetic-like nature of the particle at room temperature. The saturation magnetization of the doped samples was 91.5 emu/g for MW10ZM and 97.2 emu/g for MW15Z. These values were significantly higher than those of the reference samples MW10 and MW15, which remained at 69.3 emu/g and 82.9 emu/g, respectively. Thus, it can be concluded that, despite the small crystal size of 9.6 nm for the manganese-zinc doped sample (MW10Z) and 14.6 nm for the zinc doped sample (MW15Z), the saturation magnetization decrease can be compensated for by the doping.

**Figure 7:**
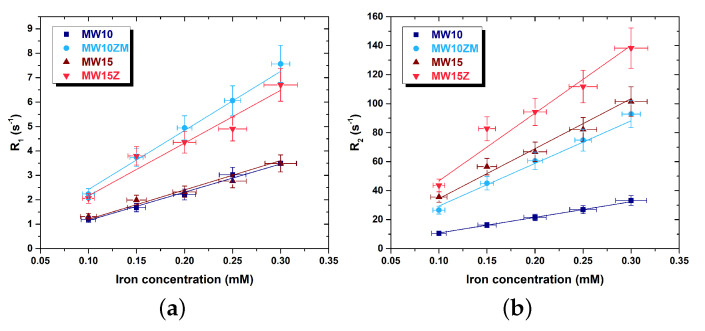
Longitudinal R${}_{1}$ (**a**) and transverse R${}_{2}$ (**b**) relaxation rates of the selected samples as a function of the iron concentration at 1.5 T.

The ${}^{1}$H-NMR measures at 1.5 T of the 1/T${}_{1}$ and T${}_{2}$ relaxation times as a function of the iron concentration are reported in Figure 7. The samples maintain their colloidal stability in water over time (Figure S5), but after 1 h from the first NMR acquisition (i.e., the first application of the static magnetic field), the longitudinal and transverse nuclear relaxation times become longer (Figures S3 and S4). As expected, from the comparison between the doped and undoped MNPs, we can see that the introduction of the doping elements increases both the longitudinal and transverse relaxivity values. The r${}_{2}$/r${}_{1}$ values reported in Table 2 show that all the samples are good T${}_{2}$ contrast agents, and, thus, the analysis can be focused on the T${}_{2}$ shorting efficiency. The zinc–manganese doped iron oxide particles of the “10 nm” size set exhibit an r${}_{2}$ value of 294 ± 44 s${}^{-1}$mM${}^{-1}$, approximately three times higher than the reference MW10 (108 ± 16 s${}^{-1}$mM${}^{-1}$). The zinc-doped MNPs show an r${}_{2}$ value of 467 ± 70 s${}^{-1}$mM${}^{-1}$, instead of the 345 ± 52 s${}^{-1}$mM${}^{-1}$ r${}_{2}$ value of the MW15 reference sample and so, in this case also, the introduction of the doping significantly enhances the relaxation rates of the ferrites.

## 4. Discussion

As previously reported, we observed that the size of the MNPs can be tuned with the nature of the polyol: polyols with longer chains such as TEG promote the growth of the MNPs more than those with shorter chains such as DEG [29]. However, this work states that the selection of metallic precursor rates also influences the growth mechanism and, consequently, the final size of the MNPs.

The progressive substitution of Fe ions with the Mn ions leads to the expansion of the lattice and the reduction of the crystal size; thus the final crystal size of the MNPs cannot be explained by a mere expansion or contraction of the lattice parameter. Instead, the XRD data suggest that the reduction of the particles’ crystal size might be traced back to the Zn and Mn ions present on their surface, which might drastically modify the surface energy, and thus the growth rate of the samples. The progressive shift of the crystallography peaks in the XRD patterns, as a function of the doping amount, is representative of the incorporation of the Mn and Zn ions in the crystal lattice instead of the formation of a new magnetic phase. In addition, the XAS results are consistent with the incorporation of the divalent cation Mn${}^{2+}$ and Zn${}^{2+}$ in the octahedral sites instead of the Fe${}^{2+}$ ions, which would preserve the spinel structure, partially doped. The unmodified pre-edge peak observed in doped samples is proof of this. When the Mn${}^{2+}$ ions are embedded in the O${}_{h}$ site, they substitute the Fe${}^{3+}$ ion and bring a greater contribution to the magnetic moment of the particles and thus to their saturation magnetization. We observed that zinc ions, which usually prefer to lie in the T${}_{h}$ site, are present in the O${}_{h}$ one, and for y ∼ 0.3 (Mn${}_{x}$Zn${}_{y}$Fe${}_{2-x-y}$O${}_{3}$), they maximize the saturation magnetization value, but for y > 0.3, they lead to a decrease of the net magnetic moment.

Nevertheless, even the smallest particles (around 10 nm) present saturation magnetization values above 90 emu/g (Table S2), reaching up to 96.9 emu/g, an outstanding value for MNPs of this size. Comparing our results to other studies, zinc manganese magnetite particles similar in crystal size present lower saturation magnetization (81 emu/g) [35], attributable to maghemite cores [36].

The influence of doping on the magnetic properties is also reflected in the NMR relaxation times, making these samples interesting candidates as T${}_{2}$ contrast agents. The ferrite samples MW10 and MW15 present relaxivity values comparable to those of similar sized maghemite nanoparticles synthesized by co-precipitation of iron(II) and iron(III) chloride salts [37,38]. The relaxation values reported in ref [39] as a function of the crystal size for magnetite nanoparticles , synthesized with high temperature thermal decomposition of Fe(oleate)${}_{3}$ (spherical NPs) [40], are lower than the MNPs MW10 and MW15 synthesized with the MW-assisted polyol method. The transverse relaxation values of the doped particles, synthesized by this route, are higher compared to those of samples synthesized by thermal decomposition [41]. In addition, microwave heating reduced the energy costs of the synthesis. The doped samples were also found to outperform the commercial contrast agent Endorem [42].

## 5. Conclusions

This work explores how the preparation of iron oxide MNPs doped with manganese and zinc is a smart strategy to reduce the size of superparamagnetic contrast agents synthesized by the MW-assisted polyol route, preserving an outstanding MRI contrast efficiency.

It has been highlighted how this cheap, fast, and green method is efficient for producing hydrophilic and small MNPs, with suitable magnetic properties, while maintaining high control on the structure of the products. This ensures the tuning of the physical–chemical properties for the field of application, e.g., contrast agents for magnetic resonance imaging.

The polyol and the doping elements involved during the synthesis process affect the dimensions of the MNPs. In particular, the insertion of doping elements such as Zn and Mn in the crystal lattice enhances the saturation magnetization values and hampers crystal growth. Moreover, although the defects at the surface level should reduce the saturation magnetization values, we observed, for the 10 nm particles, M${}_{S}$ above 90 emu/g, a surprising value for iron oxide MNPs with similar sizes. Thanks to the outstanding magnetic properties, these objects are interesting candidates for negative T${}_{2}$-contrast agents for MRI.

## Figures and Tables

**Table 1:** Elemental composition and angular position of the the peak due to the (311) crystallographic plane, crystal size, saturation magnetization, and lattice constant value of the selected samples MW10, MW10ZM, MW15, and MW15Z.

Sample	Composition	(311) Peak Position (°)	Crystal Size (nm)	Saturation Magnetization (emu/g)	Lattice Constant (Å)
MW10	γ-Fe${}_{2}$O${}_{3}$	35.656	9.3	69.3	8.345
MW10ZM	γ-Zn${}_{0.26}$Mn${}_{0.23}$Fe${}_{1.51}$O${}_{3}$	35.483	9.6	91.5	8.384
MW15	γ-Fe${}_{2}$O${}_{3}$	35.674	15.8	82.9	8.341
MW15Z	γ-Zn${}_{0.24}$Fe${}_{1.76}$O${}_{3}$	35.599	14.6	97.2	8.358

**Table 2:** Longitudinal and transverse r${}_{1,2}$ values of the selected samples resulting from the linear fit of the relaxation rates acquired as a function of the iron concentration at 1.5 T.

Sample	r${}_{1}$ (s${}^{-1}$mM${}^{-1}$)	r${}_{2}$ (s${}^{-1}$mM${}^{-1}$)	r${}_{2}$/r${}_{1}$
MW10	12 ± 2	108 ± 16	9.3
MW10ZM	24 ± 4	294 ± 44	12.7
MW15	12 ± 2	345 ± 52	31.9
MW15Z	22 ± 3	467 ± 70	17.1

## Data Availability

Not applicable.

## Supplementary Materials

The following are available at https://www.mdpi.com/article/10.3390/nano1010000/s1, Figure S1: TEM images; Figure S2: Hysteresis loops at 290 K; Figure S3: Stability vs. time (A); Figure S4: Stability vs. time (B); Figure S5: Stability vs. time (C); Table S1: Precursors amount; Table S2: Samples specifications.
