# Supplementary material for: Tailoring the Magnetic and Structural Properties of Manganese/Zinc Doped Iron Oxide Nanoparticles through Microwaves-Assisted Polyol Synthesis
Source: Nanomaterials (Basel). 2022 Sep 22;12(19):3304. doi: 10.3390/nano12193304 (PMC9565877; doi:10.3390/nano12193304)

Supporting information

Tailoring the magnetic and structural properties of Manganese/Zinc doped Iron Oxide Nanoparticles through microwaves-assisted polyol synthesis

Table S1. Precursors amount

Table S1. Amount of the precursors employed for the microwave-polyol assisted synthesis of the magnetic nanoparticles.

	Sample	Fe ac (mg)	Mn ac (mmol)	Zn ac (mg)	Zn ac (mmol)	Mn ac (mg)	Mn ac (mmol)
DEG	**MW10**_MW01	300	1.72	-	-	-	-
	MW1	275	1.58	27	0.15	-	-
	MW2	250	1.44	52.6	0.29	-	-
	MW3	225	1.3	78.9	0.42	-	-
	MW4	200	1.16	105	0.57	-	-
	MW5	252	1.49	42	0.23	-	-
	MW6	233	1.38	42	0.23	19	0.11
	MW7	215	1.27	42	0.23	38	0.22
	MW8	196	1.16	42	0.23	57	0.33
	MW9	178	1.05	42	0.23	76	0.44
TEG	MW10	251.2	1.45	42	0.23	-	-
	**MW15**_MW11	300	1.72	-	-	-	-
	**MW15Z**_MW12	275	1.58	27	0.15	-	-
	MW13	250	1.44	52.6	0.29	-	-
	MW14	240	1.38	42	0.23	19	0.21
	MW15	221	1.27	42	0.23	38	0.22
	MW16	202	1.16	42	0.23	57	0.33
	MW18	266	1.53	27	0.15	9	0.05

	MW19	258	1.48	27	0.15	18	0.1
	**MW10ZM**_MW20	249	1.43	27	0.15	27	0.15
	MW21	255.7	1.47	27	0.15	19	0.11
	MW22	236.5	1.36	27	0.15	38	0.22
	MW23	217.4	1.25	27	0.15	57.1	0.33

Table S2. Samples specifications

Here are reported the fundamental characteristics of the synthesized samples: **(i)** the elemental composition obtained from the ICP-OES analysis; **(ii)** the structural characteristics as the (311) peak position, the lattice constant and the crystal size, resulting from the XRD path studied with Bragg's law and Scherrer equation; **(iii)** the dimensions measured from the TEM pictures; **(iv)** the saturation magnetization values evaluated from the hysteresis loops acquired by the VSM, and corrected by the TGA analysis results.

Table S2. Morpho-structural and magnetic characteristics of the synthesized samples.

Sample	Composition	(311) Peak position (°)	Lattice constant (Å)	Crystal size (nm)	TEM size (nm)	Ms (emu/g)
			**DEG**			
**MW10_MW01**	γ-Fe_2O_3	35.656	8,345	9.3	7.8 ± 0.8	69.3
**MW1**	γ-$Zn_{0.2}Fe_{1.8}O_3$	35.543	8,370	6.7	4.6 ± 1.3	65.9
**MW5**	γ-$Zn_{0.4}Fe_{1.6}O_3$	35.465	8,388	9.3	6.3 ± 2.3	62.7
**MW2**	γ-$Zn_{0.5}Fe_{1.5}O_3$	35.429	8,396	9.4	7.8 ± 2.5	64.7
**MW3**	γ-$Zn_{0.8}Fe_{1.2}O_3$	35.420	8,398	9.4	7.8 ± 1.7	36.0
**MW4**	γ-$ZnFe_1O_3$	35.400	8,403	8.5	6.6 ± 1.7	8.2
**MW6**	γ-$Zn_{0.45}Mn_{0.15}Fe_{1.4}O_3$	35.443	8,393	9.9	7.3 ± 2.8	64.7
**MW7**	γ-$Zn_{0.5}Mn_{0.3}Fe_{1.2}O_3$	35.374	8,409	8.3	6.0 ± 1.4	59.4
**MW8**	γ-$Zn_{0. 5}Mn_{0.4}Fe_{1.1}O_3$	35.331	8,419	6.8	5.3 ± 1.5	49.7
**MW9**	γ-$Zn_{0.55}Mn_{0.65}Fe_{0.8}O_3$	35.355	8,413	5.4	6.8 ± 1.7	40.6
			**TEG**			
**MW15_MW11**	γ-Fe_2O_3	35.674	8,341	15.8	15.5 ± 3.6	82.9
**MW15Z_MW12**	γ-$Zn_{0.3}Fe_{1.7}O_3$	35.599	8,358	14.6	13.4 ± 3.4	97.2
**MW10**	γ-$Zn_{0.5}Fe_{1.5}O_3$	35.452	8,391	11.2	9.8 ± 2.2	86.1
**MW13**	γ-$Zn_{0.6}Fe_{1.5}O_3$	35.323	8,421	14.4	12.6 ± 2.8	82.9
**MW14**	γ-$Zn_{0. 5}Mn_{0.3}Fe_{1.2}O_3$	35.414	8,400	12.2	12.6 ± 2.4	84.8
**MW15**	γ-$Zn_{0. 5}Mn_{0. 5}Fe_1O_3$	35.437	8,395	10.9	10.6 ± 2.1	77.6
**MW16**	γ-$Zn_{0.5}Mn_{0.7}Fe_{0.8}O_3$	35.326	8,420	9.6	7.9 ± 2.2	46.7
**MW18**	γ-$Zn_{0.24}Mn_{0.10}Fe_{1.65}O_3$	35.487	8,383	11.7	10.1 ± 2.8	94.6
**MW19**	γ-$Zn_{0.25}Mn_{0.15}Fe_{1.6}O_3$	35.512	8,377	10.9	9.2 ± 1.9	94.0
**MW10Z_MW20**	γ-$Zn_{0.26}Mn_{0.23}Fe_{1.5}O_3$	35.483	8,384	9.6	7.7 ± 1.9	91.5
**MW21**	γ-$Zn_{0.28}Mn_{0.08}Fe_{1.64}O_3$	35.355	8,413	11.0	9.2 ± 2.2	96.9
**MW22**	γ-$Zn_{0.29}Mn_{0.26}Fe_{1.45}O_3$	35.339	8,417	10.4	7.7 ± 1.4	90.8
**MW23**	γ-$Zn_{0.29}Mn_{0.38}Fe_{1.33}O_3$	35.280	8,431	9.5	7.4 ± 1.6	83.6

Figure S1. TEM images

TEM pictures of the synthesized magnetic nanoparticles without citric acid coating.

DEG

MW10_MW01

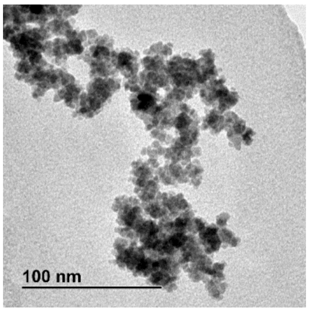

MW1

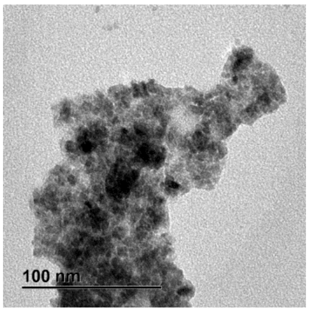

MW5

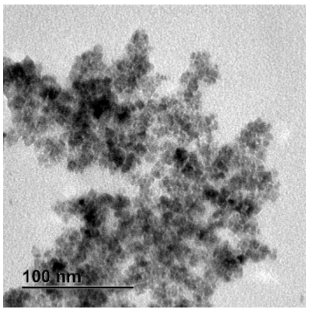

MW2

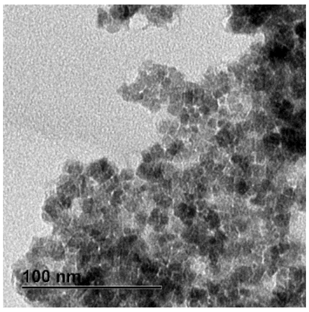

MW3

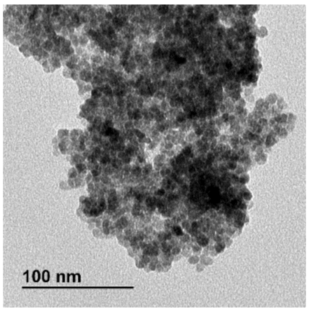

MW4

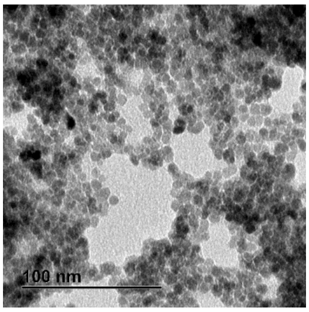

MW6

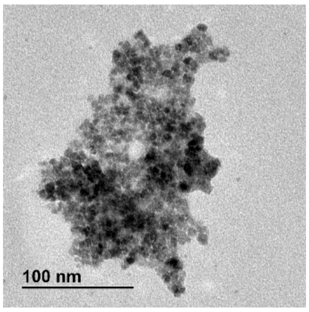

MW7

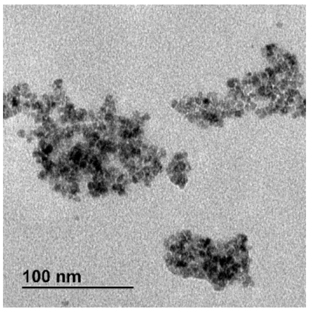

MW8

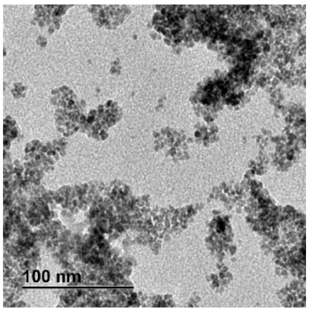

MW9

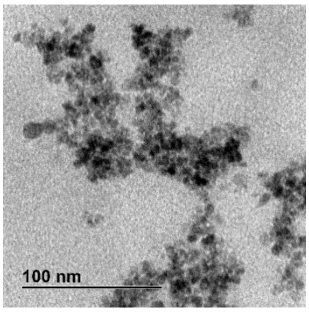

TEG

MW15_MW11

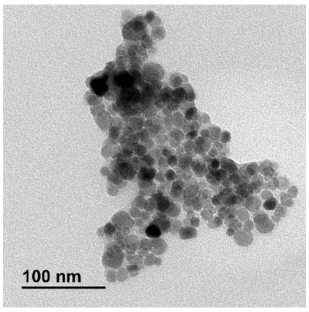

MW15Z_MW12

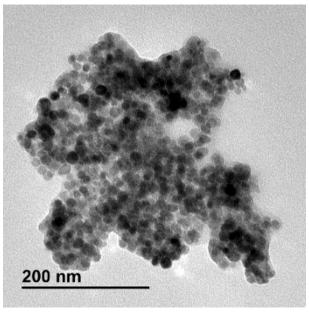

MW10

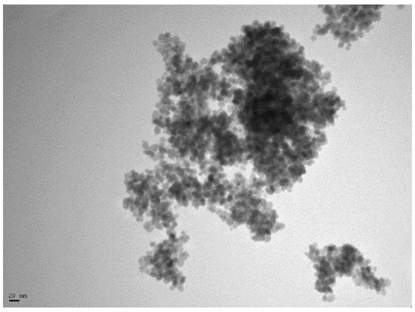

MW13

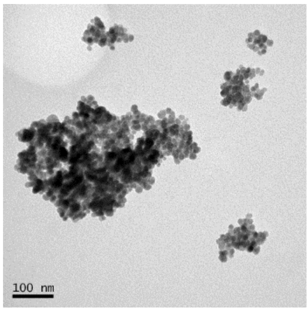

MW14

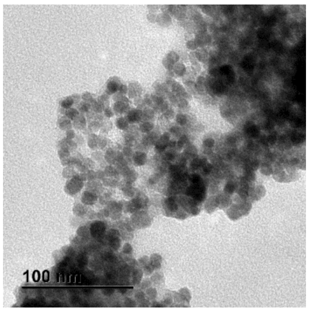

MW15

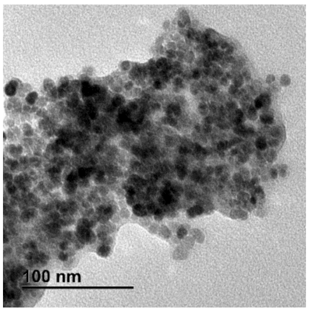

MW16

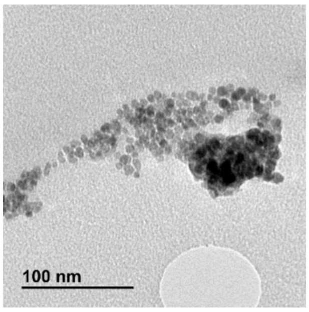

MW17

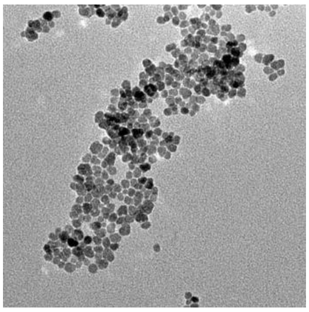

MW18

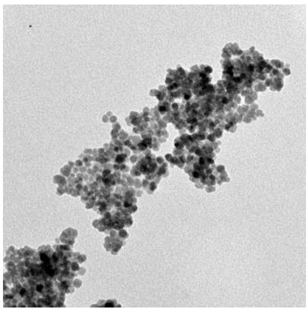

MW19

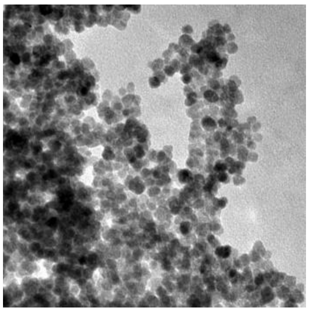

MW15Z_MW20

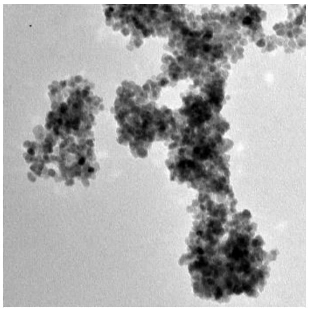

MW21

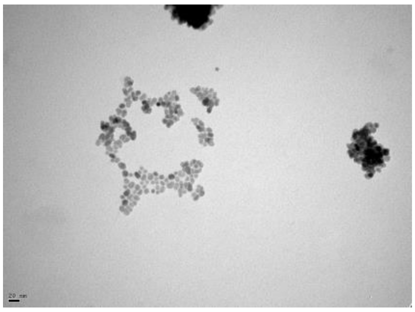

MW22

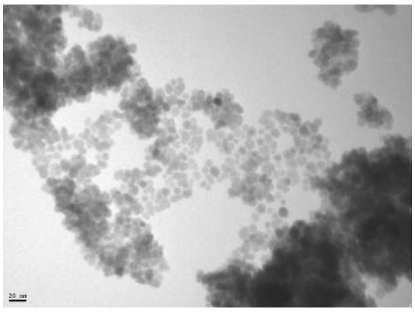

MW23

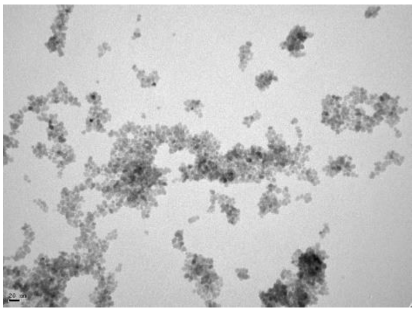

Figure S2. Hysteresis loops at 290 K

Hysteresis loops of the samples acquired at room temperature through a VSM.

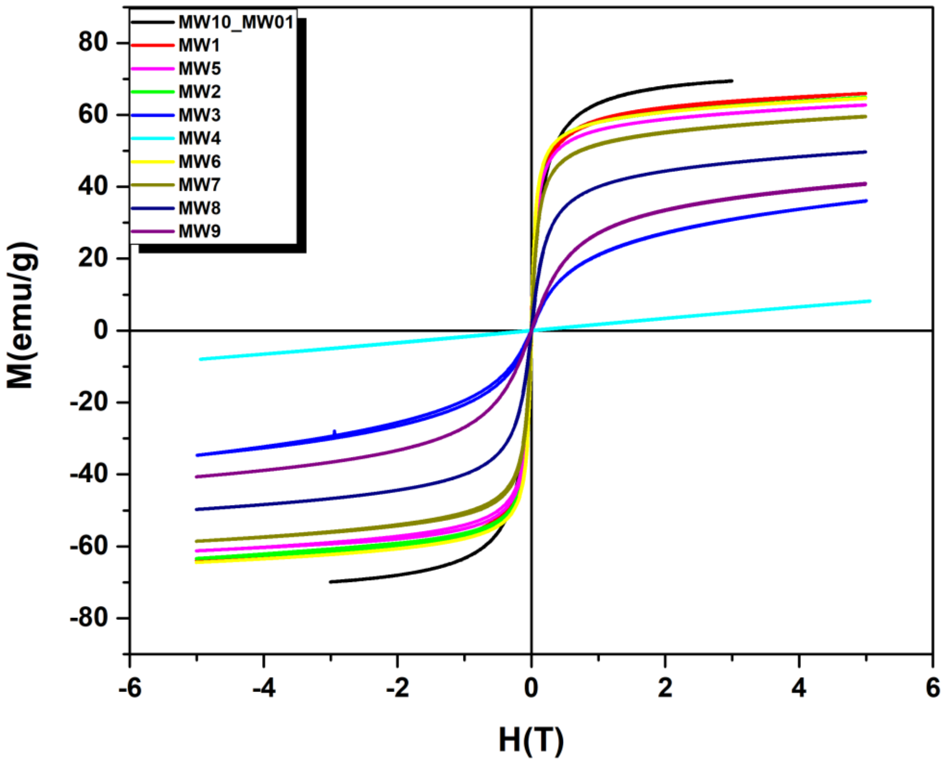

Hysteresis loop at room temperature for samples synthetized with DEG.

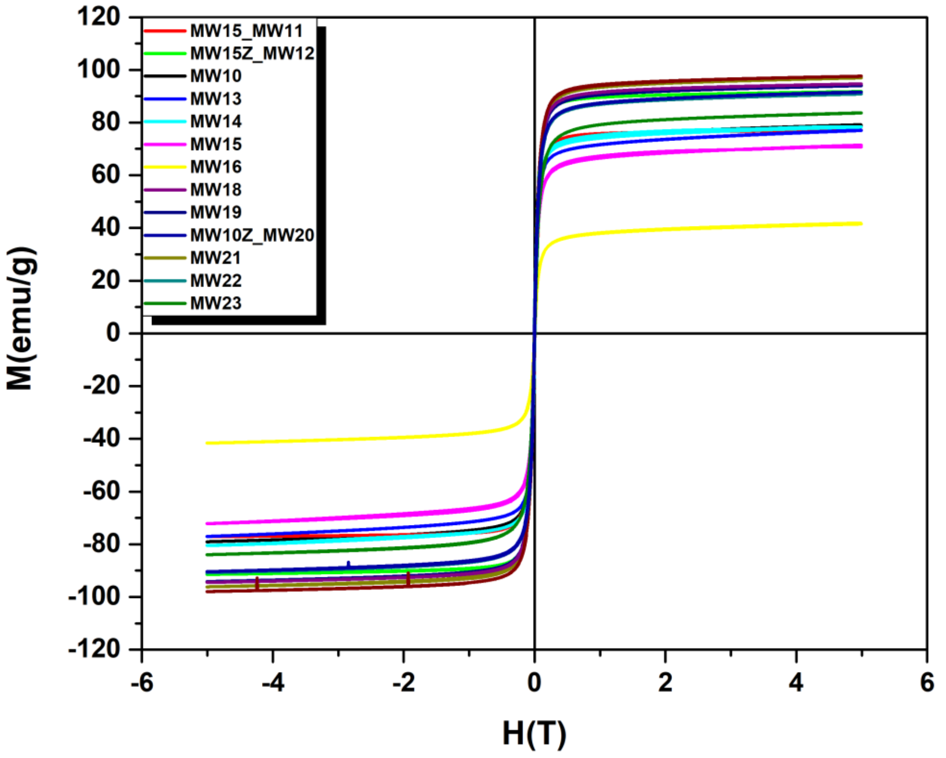

Hysteresis loop at room temperature for samples synthetized with TEG.

Figure S3. Stability vs time (A)

Here the longitudinal and transverse relaxation rates of the reference samples coated with citric acid and dispersed in 0.15 mM water solutions, measured in time. The last points are acquired after 10 minutes of sonication.

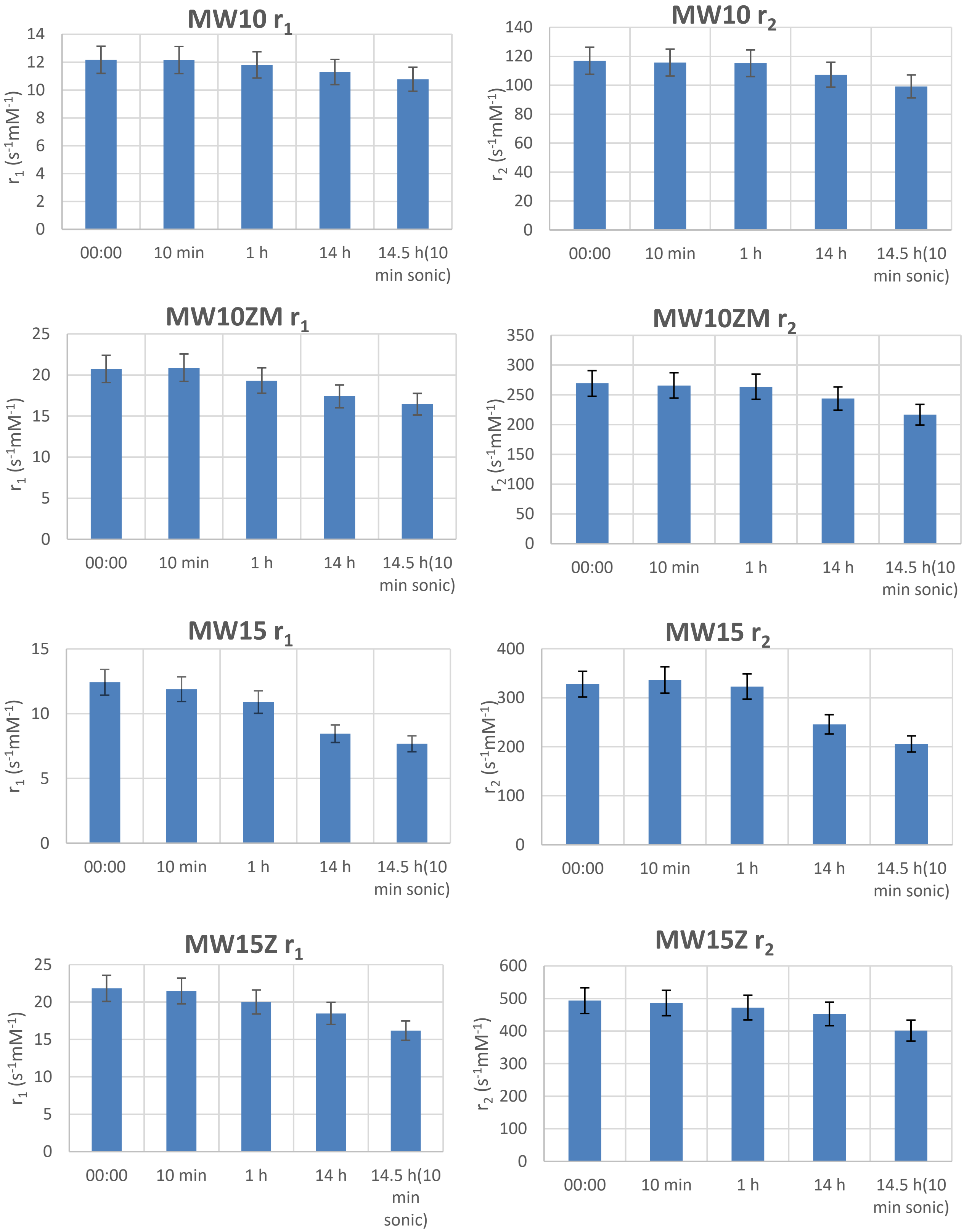

Figure S4. Stability vs time (B)

Here the longitudinal and transverse relaxation rates of the reference samples coated with citric acid and dispersed in 0.15 mM water solutions, measured in time. The last points are acquired after 2.5 minutes of sonication.

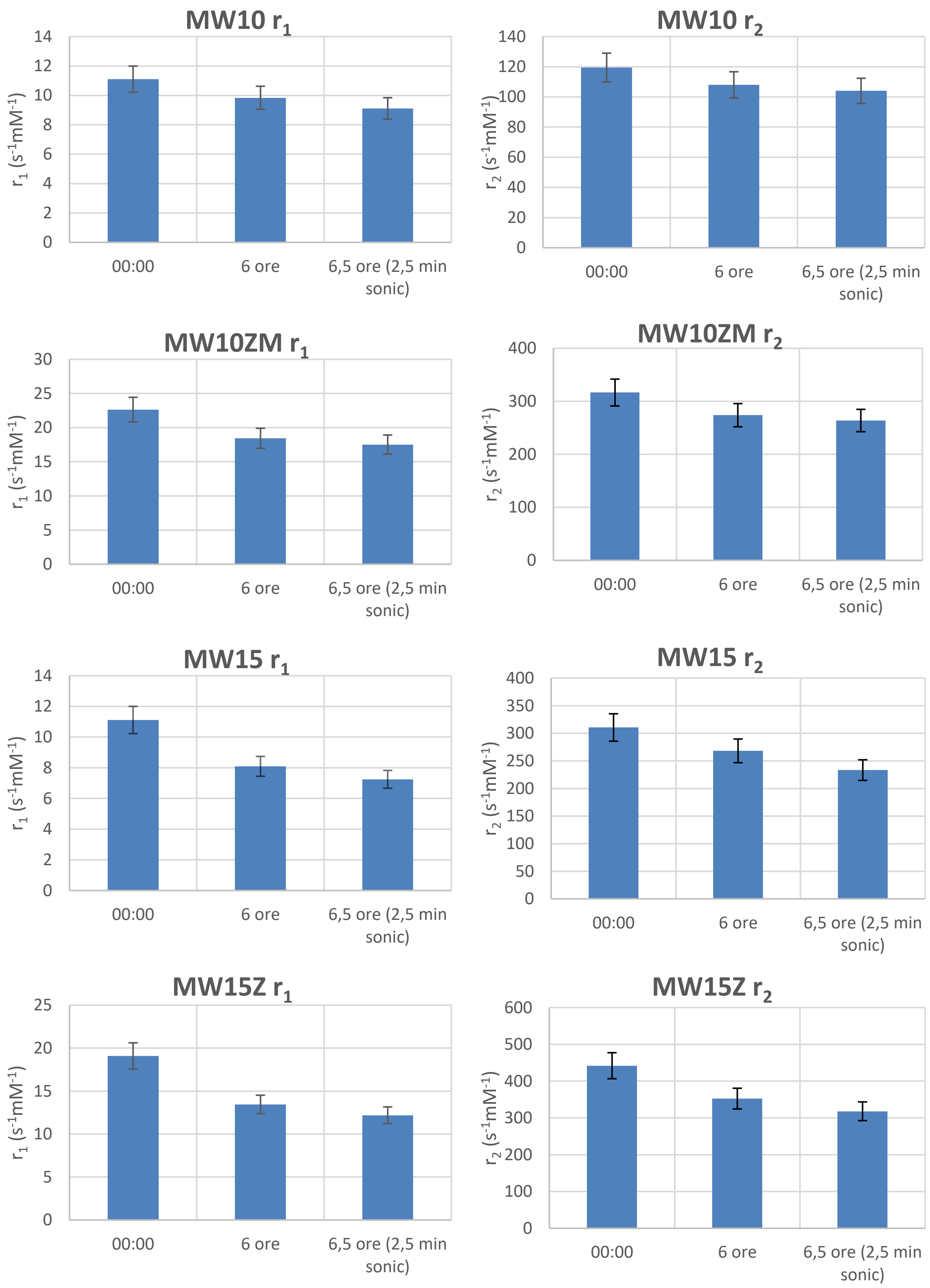

Figure S5. Stability vs time (C)

Here the longitudinal and transverse relaxation rates of the reference samples coated with citric acid and dispersed in 0.15 mM water solutions, prepared from the former solution once a month for three months.

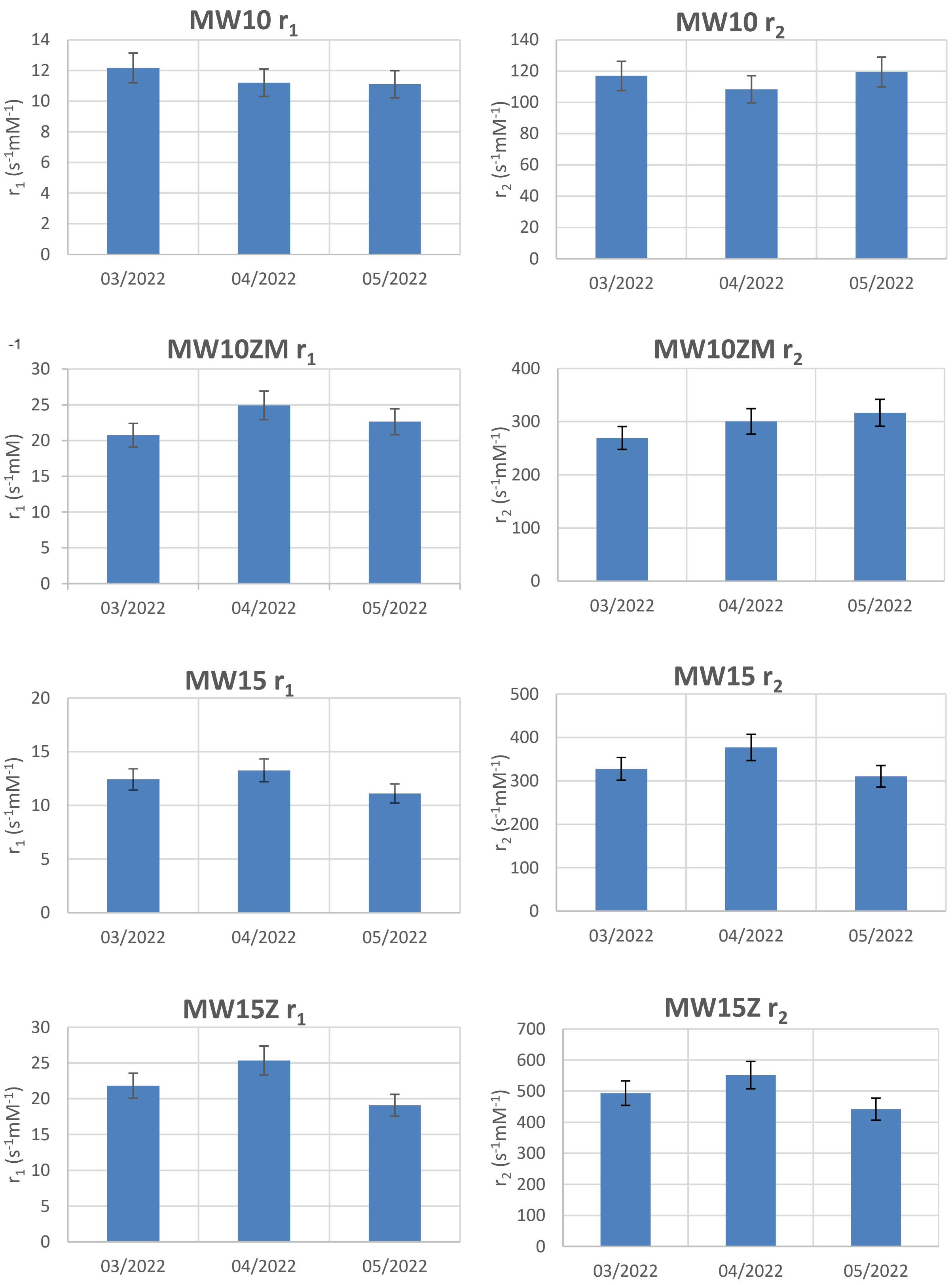

Supplement: Supplementary file 1 [file nanomaterials-12-03304-s001.zip › nanomaterials-1877840-supplementary.pdf]
